# *In vivo* imaging of *Nematostella vectensis* embryogenesis and late development using fluorescent probes

**DOI:** 10.1186/s12860-014-0044-2

**Published:** 2014-11-30

**Authors:** Timothy Q DuBuc, Anna A Dattoli, Leslie S Babonis, Miguel Salinas-Saavedra, Eric Röttinger, Mark Q Martindale, Marten Postma

**Affiliations:** The Whitney Laboratory for Marine Bioscience, 9505 N. Ocean Shore Blvd, St. Augustine, FL 32080-8610 USA; Kewalo Marine Laboratory, University of Hawaii, 41 Ahui St., Honolulu, HI 96813 USA; Molecular Cytology, Swammerdam Institute for Life Sciences, University of Amsterdam, Science Park 904, NL-1098 XH Amsterdam, The Netherlands; Université Nice Sophia Antipolis, IRCAN, UMR 7284, 06107 Nice, France; CNRS, IRCAN, UMR 7284, 06107 Nice, France; INSERM, IRCAN, U1081, 06107 Nice, France

**Keywords:** *Nematostella vectensis*, Lifeact, mTurquoise2, *EB1*, Cytoskeleton, Microvilli, Nuclear envelope, Mitosis

## Abstract

**Background:**

Cnidarians are the closest living relatives to bilaterians and have been instrumental to studying the evolution of bilaterian properties. The cnidarian model, *Nematostella vectensis,* is a unique system in which embryology and regeneration are both studied, making it an ideal candidate to develop *in vivo* imaging techniques. Live imaging is the most direct way for quantitative and qualitative assessment of biological phenomena. Actin and tubulin are cytoskeletal proteins universally important for regulating many embryological processes but so far studies in *Nematostella* primarily focused on the localization of these proteins in fixed embryos.

**Results:**

We used fluorescent probes expressed *in vivo* to investigate the dynamics of *Nematostella* development. Lifeact-mTurquoise2, a fluorescent cyan F-actin probe, can be visualized within microvilli along the cellular surface throughout embryonic development and is stable for two months after injection. Co-expression of Lifeact-mTurquoise2 with End-Binding protein1 (EB1) fused to mVenus or tdTomato-NLS allows for the visualization of cell-cycle properties in real time. Utilizing fluorescent probes *in vivo* helped to identify a concentrated ‘flash’ of Lifeact-mTurquoise2 around the nucleus, immediately prior to cytokinesis in developing embryos. Moreover, Lifeact-mTurquoise2 expression in adult animals allowed the identification of various cell types as well as cellular boundaries.

**Conclusion:**

The methods developed in this manuscript provide an alternative protocol to investigate *Nematostella* development through *in vivo* cellular analysis. This study is the first to utilize the highly photo-stable florescent protein mTurquoise2 as a marker for live imaging. Finally, we present a clear methodology for the visualization of minute temporal events during cnidarian development.

**Electronic supplementary material:**

The online version of this article (doi:10.1186/s12860-014-0044-2) contains supplementary material, which is available to authorized users.

## Background

**Figure 1:**
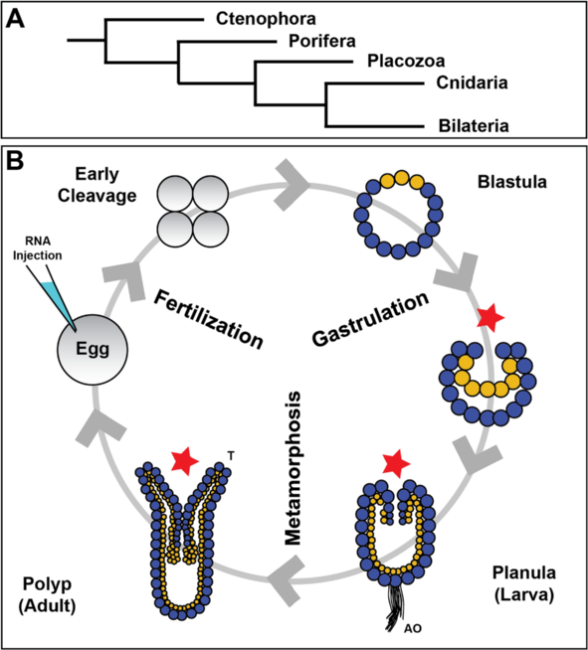
**Phylogenetic position and life-cycle of** ***Nematostella vectensis*** **. A)** Cnidarians are one of four early-evolved animal phyla that have recently been placed as the sister taxa to Bilaterians. (Phylogeny based on Hejnol [10]). **B)** The biphasic life cycle of anthozoan cnidarians like *Nematostella vectensis* begins with an early cleavage program that leads to a hollow blastula. At this stage, cells colored yellow mark the future site of gastrulation and where the mouth will eventually form (red star). During invagination the endo-mesoderm is formed, resulting in a diploblastic animal. In the ectoderm (blue) at the aboral pole of the planula larva a sensory structure called the apical organ (AO) or apical tuft forms. Once the animal undergoes metamorphosis, the animal possesses tentacles (T) used for feeding and can produce gametes for microinjection

Cnidarians and fluorescent proteins have been interconnected since the discovery of green fluorescent protein (GFP) in the jellyfish *Aequorea* [1]. Today fluorescent proteins (FP) are utilized in numerous biological processes and systems, while cnidarians have reemerged as a tool for comparative evolutionary developmental biology [2-4]. Cnidarians are the sister taxa to bilaterians (Figure 1A) and exhibit a diploblastic level of tissue organization consisting of an outer ectodermal layer and an internal endo-mesodermal layer [4]. Anthozoan cnidarians (corals, anemones and zoanthids) have a biphasic life cycle consisting of a swimming planula “larva” followed by a benthic adult polyp (Figure 1B). From the time of first cleavage, anthozoan embryos exhibit animal-vegetal polarity, where the animal pole will be the future site of gastrulation and will go on to form the mouth (Figure 1B). The uncleaved embryo is amenable to genetic tools such as transgenes [5], gene-knockdown and mis-expression [6,7] through microinjection (Figure 1B). *Nematostella vectensis* embryonic and larval/adult cell morphology have mainly been studied using Electron Microscopy (EM) or classic labeling techniques such as *in situ* hybridization or immunohistochemistry. While these traditional techniques have revealed many important aspects about the cell biology of these animals, they rely on fixed specimens and developmental processes must be inferred by imaging many embryos at specific time points of interest. Imaging using probes fused to fluorescent proteins during developmental processes is a robust technique to visualize dynamic processes in living cells and multicellular structures, with minimal introduction of artifacts created by fixation techniques [8,9]. The use of probes fused to fluorescent proteins makes it feasible to follow development in real time over a high temporal resolution and can reduce the potential artifacts that may be associated with fixation techniques.

Actin and tubulin proteins are essential for a wide range of physiological functions in many organisms, including plants and multicellular animals [11,12]. Actin is one of the most abundant proteins present within a cell, found throughout cell structures such as filopodia, lamellipodia, muscle fibrils, cell adhesion complexes, microvilli, and in the outer nuclear membrane. Actin in the nuclear membrane is associated with the nucleoskeleton and is linked to the cytoskeleton via nesprins that are part of the linker of nucleoskeleton and cytoskeleton (LINC) complexes [13]. Another abundant cytoskeletal protein is tubulin, which is present as microtubules in cilia, flagella, neuronal axons and in the mitotic spindle complex. In general both proteins play important roles during cell motility, intracellular transport, cell shape maintenance and mitosis [14-16].

In principal, the broad role of actin and tubulin in a variety of biological processes make them interesting candidates for *in vivo* visualization studies [17-19]. However, the over-expression of these proteins fused to fluorescent proteins may disrupt the normal protein function due to the size of the added fluorescent protein [18,20,21] and/or competition with endogenous proteins [22,23]. Given these limitations, probes such as Lifeact and End-binding1 (EB1) have been used for the visualization of F-actin and microtubules respectively in a number of different systems such as plants, fungi and vertebrate cells [18,23-27].

Lifeact is a 17 amino acid peptide, originally isolated from yeast, which binds with low affinity to filamentous actin (F-actin), associating with a number of actin rich cell structures such as lamellipodia, cilia and flagella [28]. Furthermore, it has been shown *in vivo* that Lifeact does not inhibit the normal function of actin or associated cellular processes [18]. End Binding protein 1 (EB1) is a plus-end-tracking protein found to act as a regulator of nucleation as well as growth of microtubules, observed in catastrophes and rescue events [29] and has been used to study microtubules dynamics [30,31].

In the following study, we used targeted probes against F-actin (Lifeact-mTurquoise2), microtubules (EB1-venus), the nucleus (tdTomato-NLS) in comparison to classical histological staining techniques to determine their versatility as structural markers for *in vivo* studies.

## Methods

### Plasmids

Coding DNA for Lifeact-mTurquoise2 was PCR-amplified from the plasmid pLifeact-mTurquoise2 N1, which was a kind gift from Joachim Goedhart. The PCR product was then cloned in pSPE3-mVenus using the Gateway system [32]. We developed two additional constructs for comparative cellular markers of the nucleus and of tubulin dynamics. A Gateway plasmid pSPE3-tdTomato (from Martindale lab) containing Nuclear Localization Signals (NLS) at the C-terminal of the fluorescent protein was utilized to identify the nucleus. A second construct was developed containing the coding region of a *Nematostella vectensis* homologue of the End-binding 1 (EB1) gene (NCBI accession #XP_001641989.1) (cloned using the forward primer – CACCATGGCCGTAA-ACGTATTTTCCACA and reverse primer - GTATTCTTCTTGTTCGAAGTCCCCG). The coding region was then sub-cloned into pSPE3-mVenus, providing the fluorescent tag at the C-terminal of the protein, using the Gateway system [32]. Finally, a Gateway plasmid pSPE3-mVenus (from Rottinger lab) was used to encode free unbound FP in the cell. Each of the four constructs was linearized and sense RNA was synthesized using the T3 Megascript kit (Ambion product #AM1338) as previously described [6].

### Microinjections

mRNA encoding the fusion protein of interest was injected into unfertilized eggs prior to fertilization as previously described [6] with the mRNA encoding Lifeact-mTurquoise2 alone and or together with either the mRNA encoding tdTomato-NLS or EB1-mVenus at the final concentration of 400 ng/μl. Live embryos were visualized approximately three to four hours after injection, when the RNA of the FP was translated into protein. Embryos were kept at room temperature (~25°C) for the first four hours of development, then transferred to an incubator set at 16°C. During imaging, embryos were at room temperature.

### Staining of fixed embryos

Cleavage stage embryos were fixed approximately 3 h post fertilization at room temperature in fresh 3.4% paraformaldehyde, HEPES 0.1 M (pH 6.9), EGTA 0.05 M (pH 8–9), MgSO_4_ 0.005 M, Dextrose 0.4 M, Triton x-100 0.2%, PBS 1× and distilled water for 1 hour at room temperature. Fixed embryos were rinsed 5× in PBT (PBS buffer plus 0.1% triton X-100). To visualize F-actin, embryos were incubated in Bodipy-FL phallacidin (Life Technologies, USA; Cat. # B-607) diluted 1:200 in PBT. Nuclei were visualized by incubation in Hoechst (Life Technologies, USA; Cat. # H3570) diluted 1:100 in PBT. Tubulin was visualized by incubation in anti-alpha tubulin (Sigma T6793) in 5% normal goat serum (in PBT). After incubation of the primary alpha tubulin antibody, animals were washed with PBT (5×) for ten minutes each wash. A secondary antibody (Alexa 594-conjugated α-mouse, Molecular Probes; Cat. # A-11005) was used at 1:500 to allow for visualization. All incubations were conducted over night at 4°C. In experiments where all three probes were visualized, animals were first incubated with the tubulin antibody, washed and incubated with phallacidin, Hoechst and Alexa 594 at the same time. Stained embryos were rinsed again in PBS (5×) and then dehydrated into isopropanol gradient 50%, 75%, 90% and 100% and mounted in Murray’s Mounting Media (MMM; 1:2 benzyl benzoate: benzyl alcohol), for visualization.

### Live imaging of *Nematostella vectensis* embryos

Images of embryos injected with Lifeact-mTurquoise2 mRNA were taken using a confocal Zeiss LSM 510 microscope using a Zeiss Plan-Neofluor, 100× oil immersion objective (N.A. 1.30). The samples were excited at 458 nm for mTurquoise2. Light was guided via D458/514 dichroich mirror to the sample. Fluorescence passed through a ~30 μm pinhole and a 475-505-nm band-pass filter for detection.

Images of embryos injected with Lifeact-mTurquoise2 and tdTomatoNLS mRNA were obtained using a confocal Zeiss 700 microscope using a Zeiss Plan-Apochromat, 63× oil immersion DIC objective (N.A. 1.40). The samples were excited at 458 nm for mTurquoise2 and 543 nm for tdTomato. Light was guided via D 405/488/555/639 dichroich mirror to the sample. Fluorescence passed through a ~30 μm pinhole and a 475-525-nm band-pass filter for mTurquoise2 detection and a 585-nm long-pass filter for tdTomato detection, in sequential mode.

Images of embryos injected with Lifeact-mTurquoise2 and EB1-mVenus or mVenus (untagged) mRNA were obtained using a confocal Zeiss 700 microscope using a Zeiss Plan-Apochromat, 63× oil immersion objective DIC (N.A. 1.40). The samples were excited at 458 nm for mTurquoise2 and 514 nm for mVenus. Light was guided via D458/514 dichroich mirror to the sample. Fluorescence passed through a ~30 μm pinhole and a 475-525-nm band-pass filter for mTurquoise2 detection and a 520-555-nm band-pass filter for mVenus detection in sequential mode.

Images of stained embryos were taken using a confocal Zeiss LSM 510 microscope equipped with a Zeiss Plan-Neofluar, 100× oil immersion objective (N.A. 1.30). The samples were excited at 561 nm for Alexa 594, 514.5 nm for phallacidin and 351 nm for Hoechst. Light was guided via a D UV/488/543/633 dichroic mirror (Chroma). Fluorescence passed through a ~30 μm pinhole and a 585-nm long-pass filter for Alexa-594 detection and a 520-555-nm band-pass filter for phallacidin detection and 385–470 band-pass filter for Hoechst detection in sequential mode.

### Electron microscopy (EM) in *Nematostella vectensis*

For scanning EM, embryos or juvenile polyps were relaxed in 7.5% MgCl_2_ before being pipetted directly onto a pre-wetted 0.2um GTTP filter (GTTP 01300, Millipore, USA) mounted in a Swinnex filter holder (Millipore, USA). MgCl_2_ solution was then replaced with standard EM fixative (4% gluteraldehyde in 0.1 M Na cacodylate buffer with 0.25 M sucrose and 0.002 M CaCl_2_, pH 7.5) and samples were fixed overnight at 4°C. Fixative was removed with three washes in 0.1 M Cacodylate buffer (with 15% sucrose) and samples were post-fixed for 1 h at 25°C in 2% Osmium tetraoxide (in 0.2 M cacodylate buffer) and dehydrated through a graded ethanol series. Filters were then dried in a Samdri critical point dryer (Tousimis, USA) following the manufacturer’s protocol. Dried filters were mounted on stubs using carbon tape and stored under desiccant prior to imaging with a Hitachi S-4800-I field-emission scanning electron microscope at the University of Hawaii’s Biological Electron Microscopy Facility (UH BEMF). For transmission EM, samples were collected in 1.5 ml sterile microcentrifuge tubes and were otherwise fixed as described above. Following the ethanol dehydration series, samples were washed three times (30 mins each) in propylene oxide and infiltrated in a 50:50 mix of propylene oxide and Lx-112 resin (Ladd Research Industries/Fisher Scientific, USA) overnight at 25°C, following the manufacturer’s protocol. Polypropylene/resin mix was then replaced with a fresh sample of resin three times the following day (at approximately 6 h intervals) before samples were transferred to silicone molds and polymerized at 60°C for approximately 72 h. Thin sections (~60 nm) were cut using a PowerTomeXL ultramicrotome (RMC Products, USA) and mounted on formvar-coated copper slot grids. For visualization, grids were counter-stained in 5% uranyl acetate and 0.3% lead citrate using standard procedures and imaged on a Hitachi HT7700 (UH BEMF).

## Results

### Cellular distribution of Lifeact-mTurquoise2, EB1-mVenus, tdTomato-NLS and mVenus in early cleavage embryos

**Figure 2:**
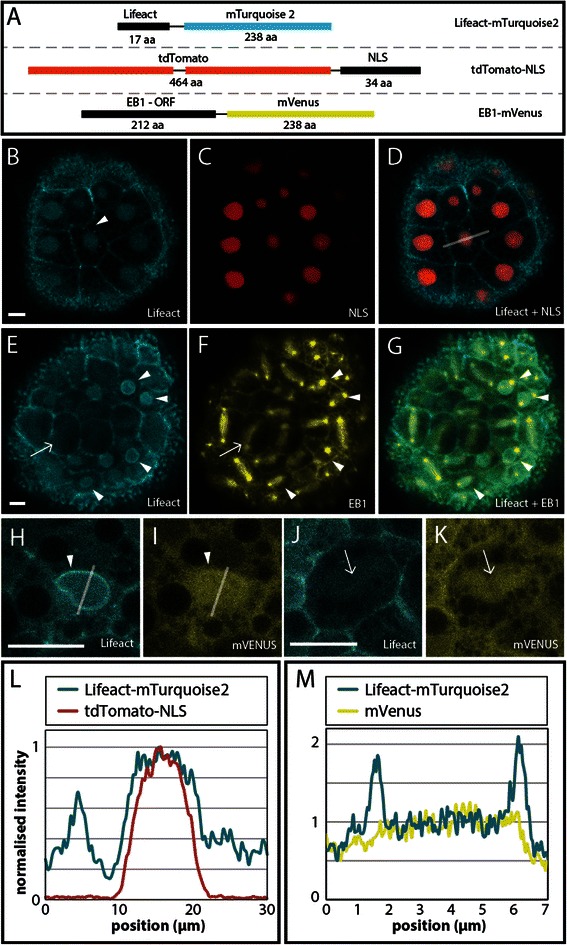
**Localization of Lifeact-mTurquoise2, tdTomato-NLS and EB1-mVenus in early cleavage embryos. A)** Messenger RNA for three different fluorescent probes was injected in pairs into embryos of *Nematostella vectensis*. **B-D)** In the same embryo Lifeact-mTurquoise2 is localized to cell boundaries and overlaps with tdTomato-NLS around the nucleus. **E-G)** In dividing cells EB1-mVenus is clearly localized at mitotic spindle fibers and centrosomes. Interestingly Lifeact-mTurquoise2 **(E)** exhibits a brighter fluorescent ring around the nucleus in a subset of cells (white arrowhead) that start to form centrosomes adjacent to the nucleus **(G)**. Lifeact-mTurquoise2 is also visible as striations corresponding to the location of spindle fibers labeled with EB1-mVenus (white arrows in **E** and **F**). **H-I)** Double expression of Lifeact-mTurquoise2 and mVenus in *Nematostella* embryos shows high concentration of Lifeact-mTurquoise2 around the nucleus **(H)** compared to mVenus used as a control **(I)** (white arrowheads). **J-K)** Both Lifeact-mTurquoise2 and mVenus appear as striations with the same morphology as spindle fibers (white arrows). **L)** Quantification of fluorescent i3ntensity of Lifeact-mTurquoise2 and tdTomato-NLS in nuclei of early cleavage stage embryos (the x-axis is represented as a white line in **D)**. **M)** Quantification of fluorescent intensity of Lifeact-mTurquoise2 and untagged mVenus in nuclei of early cleavage stage embryos (the x-axis is represented as a white line in **H** and **I)**. See main text for a more detailed description. (Scale bar =10 μm in each image)

To image early cleavage stage cellular dynamics *in vivo*, we co-injected different combinations of three chimeric constructs (Lifeact-mTurquoise2, EB1-mVenus and tdTomato-NLS; Figure 2A) (see Methods and Figure 1B)*.* Fluorescence from all three translated proteins was first detectable approximately 2–3 hours after fertilization. Expression of Lifeact-mTurquoise2 is visible throughout the cytoplasm during early embryogenesis, but exhibits increased fluorescence at cell boundaries, consistent with the cortical F-actin (Figure 2B, white arrowhead). The cytoplasm also exhibits distinct dark areas that lack fluorescence, which are likely to be platelets of yolk abundantly present in early stage *N. vectensis* embryos. tdTomato-NLS predominately localizes to nuclei apart from a narrow band at the nuclear edge that completely overlaps with nuclear Lifeact-mTurquoise2 (Figure 2C-D). In a subset of cells, potentially at a different stage of cellular division, (Figure 2E, white arrowhead) Lifeact-mTurquoise2 is visible as a ring around the nucleus. These embryos were co-injected with EB1-mVenus, which localizes to both centrosomes and spindle fibers (Figure 2F-G). The cells that exhibited increased levels of Lifeact-mTurquoise2 around the nucleus (Figure 2F-G, arrowheads) have yet to completely form spindle fibers (visualized by EB1-mVenus). In control samples (Figure 2H-K) that were injected with Lifeact-mTurquoise2 and mVenus, cells exhibiting heighted nuclear mTurquoise2 also have ubiquitously expressed mVenus (Figure 2H-I). One common result that was observed with each cytoplasmic present FP, is the localization of fluorescence in and/or around the spindle fibers (Figure 2E-F, 2J-K, white arrow). It is our understanding that this signal, or faint localization, is a non-specific localization (see discussion for details).

We quantified the fluorescence intensity throughout a cell (Figure 2D, white line) and found that Lifeact-mTurquoise2 fluorescence around the nucleus is significantly higher than the fluorescence in the cytoplasm (Figure 2L), however the localization of fluorescence in the nucleoplasm is likely non-specific because it is also found in the control using mVenus (Figure 2M). The quantification of tdTomato-NLS in the same cell shows that signal is directly overlaps the expression of nuclear Lifeact-mTurquoise2 (Figure 2L). Quantification of nuclear fluorescence intensity in control embryos expressing Lifeact_mTq2 and mVenus indicates that Lifeact_mTq2 is enriched around the nuclear boundary, where mVenus exhibits little change along the same measurement (Figure 2H-I,M).

### During embryonic cleavage, a distinct accumulation of Lifeact-mTurquoise2 localizes to the nuclear boundary

During early development in *Nematostella vectensis,* the embryo undergoes approximately eleven cleavage cycles to reach blastula stage [28], which can be visualized in embryos injected with Lifeact-mTurquoise2 and EB1-mVenus (see Additional file 1: Video S1, Additional file 2: Video S2 for a time series of this process, revealing all the events occurring during cell division in *N. vectensis* embryos in real time).

**Figure 3:**
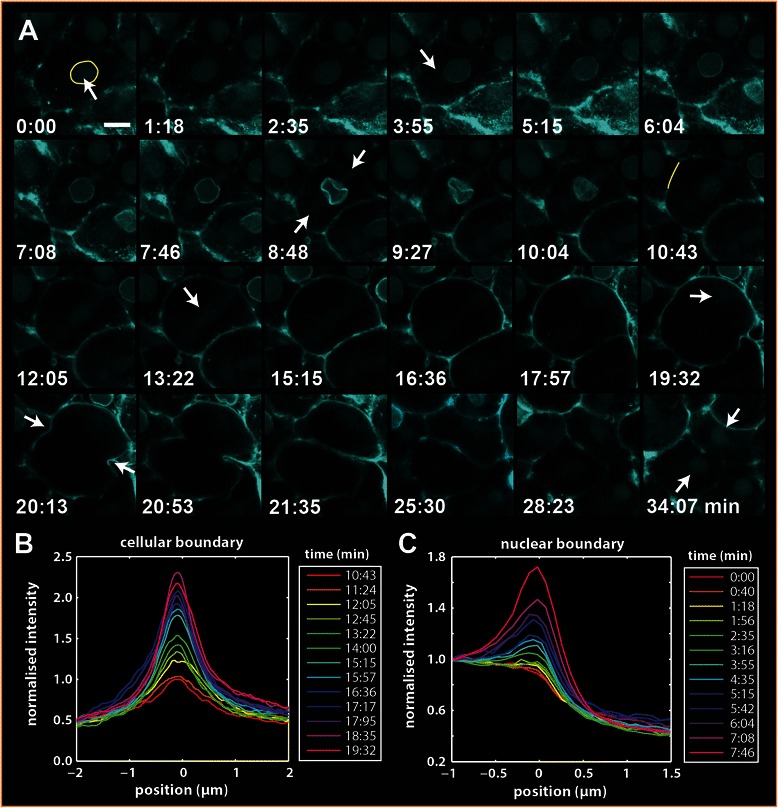
**Lifeact-mTurquoise2 localizes to the nuclear boundary and exhibits a ‘flash’ of Lifeact during nuclear disassembly. A)** Time series of a living cell undergoing cell division. Just prior to nuclear disassembly, accumulation of Lifeact is apparent (A2.0-6.0) and during prometaphase disappears with a ‘flash’ (A6.5-7.5). See main text for a more detailed description of the full cleavage cycle. Scale bar 10 μm. **B)** When the cell rounds up during metaphase-anaphase Lifeact-mTurquoise2 fluorescence appears to increase at the cell boundary through time (different time points are indicated by colored lines). The average profiles perpendicular to the plasma membrane region going from the intracellular space (left) to the extracellular space (right) from the region depicted in A8.0 (yellow line) are normalized to the peak value of the profile at A8.0, show for this cell an increase of about 2.5 fold. **C)** Quantification of the Lifeact fluorescence at the nuclear boundary from 0.0-6.0 min. The profiles show the averaged normalized fluorescence perpendicular to the nuclear boundary going from nucleoplasm (left) to the cytoplasm (right)

To better characterize cellular dynamics of Lifeact-mTurquoise2 during a cleavage event, we concentrated on visualizing individual cells with sufficient zoom and time resolution (Figure 3, Additional file 3: Video S3a, b). In this time series (images were captured approximately every 30–40 seconds) we were able to observe several distinct phases of the *N. vectensis* cell cycle. Figure 3A shows an image sequence of one typical cell prior to (Figure 3A, 0:00) and during division (Figure 3A, 20:13 arrows indicate axis of division). At 2:35, we first observe an accumulation of Lifeact at the nuclear boundary and at the same time, centrosomes become visible as regions of increased cytoplasmic Lifeact-mTurquoise2 which form adjacent to the nucleus (Figure 3A, 3:55 arrow). As we previously stated, the non-specific labeling of spindle fibers and centrosomes appearing in FP injections, can be used to identify these structures. The accumulation of Lifeact-mTurquoise2 in a ring around the nucleus continues over a time course of about 4 minutes. After this phase (reminiscent of prophase), we observe a distinct deformation of the circular structure around the nucleus (Figure 3A, 8:48 arrows), which starts at the position of the presumptive centrosomes and often results in a donut shaped nucleus. (also see Additional file 1: Video S1A, Additional file 3: Video S3a). During this phase, we observe that the centrosomes move further away from the nucleus and that in the time course of about two minutes the nucleus disassembles, during which the nucleus elongates and a fibrous network of Lifeact is visible (Figure 3A 13:22).

After complete disassembly, Lifeact-mTurquoise2 is visible as striations in the cytoplasm (Figure 3A 13:22, arrow) as previously discussed in Figure 2. The cell gradually assumes a spherical shape during metaphase and anaphase (Figure 3A 10:43–17:57), which is often associated with an increase in Lifeact-mTurqoise2 fluorescence at the plasma membrane (Figure 3A 10:43–19:32), which is not observed in mVenus control injections (Additional file 4: Figure S1B). This phenomenon was observed in many cells and in different embryos over time (see Additional file 4: Figure S1A). The quantified increase of Lifeact-mTurqoise2 fluorescence at the plasma membrane is shown in Figure 3B, where peak normalized average profiles across the plasma membrane are shown for the region depicted in Figure 3A, 10:43 (yellow line). Based on 12 cells from different embryos we find that this increase is variable with an average of 1.6 fold and 95% confidence interval of [1.3, 1.9] (Additional file 4: Figure S1C). The centrosomes move in opposite directions close to the plasma membrane (Figure 3A 19:32); at the same time, the cleavage furrow starts to form and becomes visible (Figure 3A, 20:13 arrows). The cells are fully cleaved within 2 minutes thereafter. The nuclei in the daughter cells are then reassembled and are fully visible again after about 8 minutes (Figure 3A, 34:07 arrows). A complete cleavage event, starting from the increase of nuclear Lifeact-mTurquoise2 to reappearance of the nuclear Lifeact-mTurquoise2 in the daughter cells takes approximately 32 minutes (Figure 3A, 2:35–34:07, Additional file 3: Video S3B).

To further quantify the transient increase of the Lifeact (‘flash’) near the nuclear boundary, we constructed a contour around the nuclear edge for each time point. The average intensity profile perpendicular to the nuclear boundary, normalized with respect to the nucleoplasm intensity was then calculated. The profiles from the cell shown in Figure 3A reveal that Lifeact-mTurquoise2 undergoes an approximate two-fold increase in fluorescence that commences about 4 minutes before deformation and disassembly of the nucleus (Figure 3C). Based on 16 cells from 3 different embryos we found an average increase of 2.1 fold, with 95% confidence interval of [1.9, 2.2] (Additional file 4: Figure S1D). Analysis of the time series suggests that the accumulation of Lifeact-mTurquoise2 localizes around the outer surface of the nucleus in a greater quantity that what is found in the nucleoplasm (Additional file 3: Video S3a, Additional file 5: Video S4). Throughout early development, virtually all cells exhibited a Lifeact ‘flash’ if imaging was conducted at the correct time during division.

**Figure 4:**
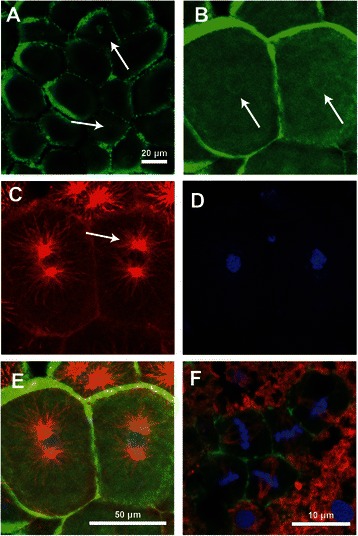
**Labeling of F-actin, DNA and tubulin in fixed embryos. A-B)** Preserved cells labeled with the F-actin stain, phallicidin-FL (green) exhibit F-actin at the nuclear boundary of cells that are at an early phase of cell division. **C)** Alpha-tubulin staining (red) shows the formation of centrosomes adjacent to the nucleus. **D)** DNA staining with Hoechst (blue) show that the nuclear structures are still intact. **E)** Overlay of all three channels from **B-D** shows the relative position of all markers. **F)** Several cells at metaphase stained with the same labels show the fully formed mitotic spindles and the chromosomes that are lined up along the metaphase plate. F-actin associated with the nuclear boundary is no longer present, but is faintly visible in the cytoplasm and clearly at the plasma membrane

The ‘flash’ of Lifeact at the nuclear boundary doubles in intensity with respect to background fluorescence in the nucleoplasm and is distinctly visible during a relatively short time window of approximately two minutes. To further validate and corroborate the results found with Lifeact-mTurqoise2 we used classical staining techniques in preserved embryos. Similar to Lifeact-mTurquoise2 labeled embryos, phallacidin staining labels F-actin near the nuclear boundary (Figure 4A-B, white arrows). Co-labeling with anti-tubulin reveals that it is in the cells in which centrosomes are beginning to form that the nuclear boundary exhibits F-actin staining (Figure 4B-C, white arrows). In these cells, centrosomes with tubulin containing spindle fibers are forming on either side of the nucleus (Figure 4D,E) at a time when the DNA of the nucleus is still compartmentalized (Figure 4D). In cells where spindle fibers have formed, F-actin appears brightest along the cellular boundaries (Figure 4F). These results corroborate the observations obtained from Lifeact-mTurqoise2 imaging, and suggest that the accumulation of Lifeact-mTurqoise2 at the nuclear boundary occurs prior to the onset of nucleus deformation and breakdown at prometapahase.

### Lifeact-mTurquoise2 a suitable marker for monitoring cell shapes in multicellular embryos

**Figure 5:**
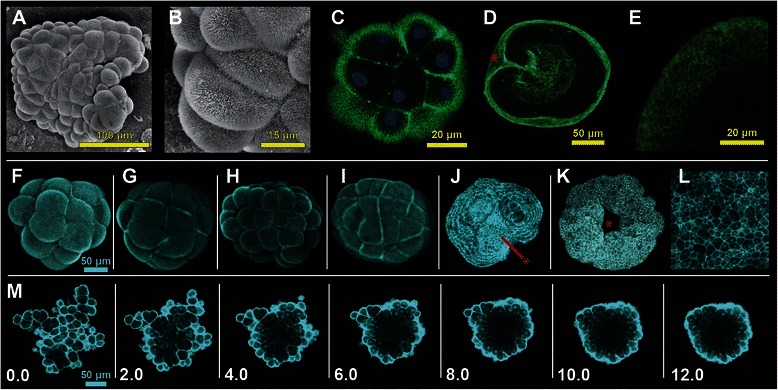
**Localization of Lifeact-mTurquoise2 in microvilli and cell boundaries. A-B)** Preserved embryos at early cleavage stages visualized using scanning electron microscopy, show microvilli on the cell surface. **C)** Similar stage preserved embryos stained using phallacidin to highlight filamentous-actin containing microvilli at the cells surface. **D-E)** During gastrulation, phallacidin can be used to visualize cellular boundaries along the outer surface of the gastrula. **E)** Zoomed in region of the ectoderm showing cellular boundaries. **F-I)** Cleavage stage embryos injected with the mRNA of Lifeact-mTurquoise2. **F-G)** Embryo approximately at the 32-cell stage, exhibits an increase in surface area contact between neighboring after cell cleavage. **H-I)** Embryo approximately at the 64-cell stage exhibits an increase in surface area contact between neighboring cells after cell cleavage. **J-L)** Gastrula stage embryos labeled through injection of Lifeact-mTurquoise2 RNA. **J)** Early gastrula where the endodermal plate is clearly visible by Lifeact-mTurquoise2 protein (red asterisk). **K)** Late gastrula where the endodermal plate has invaginated inward out of view (red asterisk – site of gastrulation). **L)** Magnification of cell boundaries clearly labeled with Lifeact-mTurquoise2 from late gastrulation stage embryo shown in **K**. **M)** Time series of an early embryo with a loose aggregate of cells with a flattened configuration that develops into a compact ball-shaped embryo, thereby increasing cell-cell contact (0.0-12.0 min, Additional file 7: Video S6)

F-actin can be detected in microvilli, protrusions of the cellular membrane [33,34]. In *N. vectensis,* these structures can be visualized by scanning electron microscopy during early embryonic development (Figure 5A,B). A similar structure is visible by confocal microscopy of early embryos labeled with the F-actin binding molecule, phallacidin (Figure 5C). Phallacidin labeled embryos during gastrulation highlight the separation of ectodermal and endodermal tissue (Figure 5D) and distinguishes cell boundaries along the ectodermal surface (Figure 5E). During early embryogenesis, Lifeact-mTurquoise2 localizes to microvilli along the cell surface, and highlights cellular boundaries providing a three dimensional shape when confocal images are stacked from images taken along the z-axis (Figure 5F). During early development Lifeact-mTurquoise2 can be used to visualize microvillar actin bundles along the cellular surface, which exhibit erratic swirling behavior along the cellular boundary (see Additional file 6: Video S5). Immediately prior to cleavage events, we found that embryos become more spherical in shape up to the point of cleavage at which the cells lose their round shape and appear to increase adjacent cell-cell contact (Figure 5F vs. G,H vs. I). Embryos in Figure 5F-G or H- I were taken from a time series of cleavage events, from very early development (approximately 32–64 cell stage). Embryos in F and H were taken after embryos that had recently divided, while G and I are what the embryo looks like before undergoing division. Generally, prior to division, the embryo as a whole exhibits a more compact or round shape with increased surface contact between neighboring cells than earlier stages with roughly equal numbers of cells (F and H, respectively). Although cell counts were not performed, during this phase of development, it has been reported that cell division occurs in a highly synchronous fashion [35].

We found that Lifeact-mTurquoise2 allows for visualization of cellular shape changes in multicellular embryos through the visualization of the F-actin enriched microvilli and at the cell-cell interfaces during embryogenesis. We were able to clearly visualize cellular boundaries during cleavage stages and gastrulation (Figure 5J-L), where the endodermal plate (Figure 5J) moved inside the blastopore (Figure 5K). Figure 5M shows an early stage embryo that forms a compacted ball from a loosely packed cluster of cells over a time course of about 12 minutes. (see also Additional file 7: Video S6). The cells appear to increase cell surface contact and are pulled into the ball, similar to a process where cell-cell adhesion increases. These data show that Lifeact-mTurquoise2 can be used as a cell surface and boundary marker to follow developmental processes.

### Lifeact reveals a broad range of adult *Nematostella vectensis* specific cell-types and cell-structures

**Figure 6:**
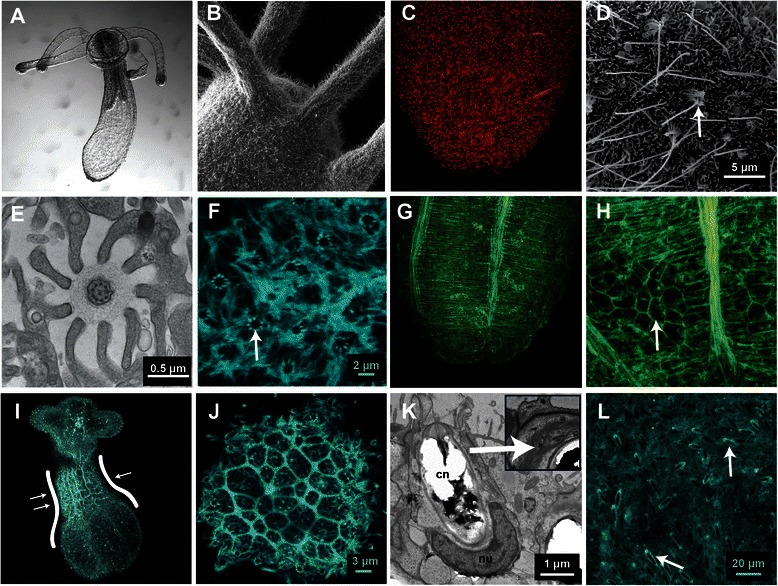
**Lifeact-mTurquoise2 protein can be used to identify many different structures in adult** ***Nematostella vectensis*** **. A)** DIC image of a juvenile polyp approximately two weeks after fertilization. **B)** SEM image of the oral portion of the animal showing the ciliated cells at the surface. **C)** Laser confocal microscopy stack image of the aboral end of the polyp labeled with anti-alpha tubulin (secondarily labeled with Alexa 594) showing the tubulin that is present in ciliated cells. **D)** SEM image of the body wall of a polyp showing long cilia (white arrow) surrounded by ciliary cones. **E)** TEM image of a single central cilium with typical 9 + 2 arrangement of microtubules and nine stereocilia surrounding it. **F)** Body wall of a polyp originally injected during embryogenesis with Lifeact-mTurquoise2. Lifeact-mTurquoise2 highlights the stereocilia structures (white arrow) identified in **D**,**E**. **G-H)** Phallacidin labeled fixed polyp showing the intricate network of F-actin in the muscular tissue within the body **(G)** as well as cellular boundaries (**H**, white arrow). **I)** Single image from a time series of a moving polyp exhibiting an increased level of Lifeact-mTurquoise2 at the site of contraction (white arrows, also see Additional file 8: Video S7.) **(J)** Lifeact-mTurquoise2 also binds to the actin found in cell boundaries in polyps, similar to phallacidin **(H)**. **K)** TEM image of cnidocyte (cn) with a curved nucleus at the basal pole (nu) showing filamentous actin-like fibers around the apex of cnidocyte capsule (inset). **L)** Prominent Lifeact-mTurquoise2 fluorescence is present around the apical and lateral regions of the cnidocytes, suggesting F-actin is present around the perimeter (white arrows)

F-actin is a core component of a variety of different cellular structures and therefore is broadly distributed throughout an animal. We used the adult polyp stage (Figure 6A), which exhibits many differentiated cell-types, to determine what structures are enriched with F-actin and can be reliably visualized *in vivo* using Lifeact-mTurquoise2.

The outer surface of the polyp is covered with ciliated cells, including the oral region (Figure 6B) and these cells contain microtubule fibers detectable with tubulin antibody staining (Figure 6C). A closer look at the structure of the cilia reveals that each long cilium is surrounded by a ring of microvilli (Figure 6D) forming a ciliary cone structure. A transmission electron micrograph through a single cilium reveals the dense structure of the cilium, containing two microtubule fibers at the center and nine surrounding doublets that originate from the centriole; the cilium is surrounded by nine microvilli (Figure 6E). An adult injected with Lifeact-mTurquoise2 reveals that Lifeact is enriched at surface microvilli and distinctly at ciliated cells that exhibit a pattern associated with the nine microvilli that accompany each cilium (Figure 6F), similar to Figure 6D-E.

The muscular system (Figure 6G) as well as cell boundaries (Figure 6H) are rich with F-actin and can be visualized using Phallacidin. As an adult, animals move through ciliary action and peristaltic movements (Additional file 8: Video S7). These movements are a result of muscular contraction and are accompanied by an increase in Lifeact-mTurquoise2 fluorescence around the region of constriction (Figure 6I). Similar to Phallacidin stained animals, the cell boundaries are clearly visible in animals injected with Lifeact-mTurquoise2 (Figure 6J).

Cnidocytes are a cnidarian specific cell-type, and are thought to be derived from neural stem cells [7,36]. A transmission electron micrograph through a cnidocyte at the ectodermal surface shows filamentous structures reminiscent of F-actin around the apex of the unfired cnidocyte capsule (Figure 6K). An adult, that was injected with Lifeact-mTurquoise2 as an embryo, reveals that Lifeact also exhibits polarized fluorescence at the apex of the cnidocyte (Figure 6L) confirming these regions are enriched with F-actin.

### Fluorescent properties of mTurquoise2 allow for long exposure *in vivo* visualization with minimal toxicity

The fluorescent protein mTurquoise2 exhibits one of the highest quantum yields and is relatively photo-stable making it bright and resistant to photo-bleaching [37]. To determine the versatility of this fluorescent protein as a structural marker during development (without toxicity or bleaching), we took z-stack time-lapse images for six hours, at six different focal planes and for every ten minutes (Additional file 9: Figure S2A). A total of 144 scans per embryo (*N* = 5) were obtained to determine to what extent long-term visualization would have an effect on normal development of the animal (Additional file 9: Figure S2B). All animals were kept after each imaging session and developed to the polyp stage (data not shown). Overall, long-term exposure analysis of Lifeact-mTurquoise2 during embryonic development did not appear to result in noticeable developmental defects. Lifeact imaged embryos did not appear to lose fluorescent intensity over the long visualization period, where visualization of tdTomato or mVenus exhibited rapid bleaching over time (data not shown). Lifeact was still visible two months after injection of Lifeact-mTq2 mRNA into uncleaved eggs, long into the juvenile phase. Summarizing, and maintains a similar cellular localization as in early juveniles (Figure 6I-J,L). mTurquoise2 is an excellent fluorescent protein for imaging embryogenesis, allowing rapid and dynamic cellular processes (e.g. cleavage division) to be captured, while at the same time enabling long imaging sessions, with low photo-bleaching and limited photo toxicity.

## Discussion

In this study, we used transient expression of Lifeact-mTurquoise2 in conjunction with other fluorescent probes and cellular markers for the visualization of developmental phenomenon of the cnidarian *Nematostella vectensis*. These methods allow for imaging of cellular boundaries, microtubule dynamics and individual cell-type identification during development. The use of fluorescent probes such as the Lifeact- mTurquoise2 and EB1-mVenus used here enhances *in vivo* research of biological processes and can aid in phenotypic studies of gene knock-down. More specifically dynamic aspects of cell cleavage, early gastrulation as well as wound healing could be studied in much more detail.

### mTurquoise2 tagged probes, a bright way to follow development in *Nematostella vectensis*

During early stages of development in *N. vectensis*, yolk is present throughout the cytoplasm, which limits imaging of fluorescent proteins deep into the embryo. High laser power can be used to obtain sufficient signal but this also leads to increased photo-bleaching. We observe that fusion of probes to mTurquoise2 indeed allowed for imaging (~2 hr, 1–2 fpm), with little photo-bleaching (<10%) and without any detectable photo-toxicity. We attempted similar long-exposure studies with tdTomato-NLS and Eb1-mVenus but bleaching of the fluorescence protein limited the length of time over which data could be collected (~50% bleaching of mVenus in 2 hour, for example see Additional file 1: Video S1, Additional file 2: Video S2). This study has shown that mTurquoise2 greatly improves live imaging in *Nematostella vectensis.*

### Visualization of F-actin and tubulin dynamics during *Nematostella vectensis* cell division

By using a combination of Lifeact and EB1 we were able to observe cleavage events in real time. It is our current hypothesis that the process of cell division in *Nematostella vectensis* occurs through open mitosis, also found in many bilaterian taxa [13]. During open mitosis, the Nuclear Envelope breaks down, releasing the DNA for segregation into daughter cells. Prior to spindle fibre formation in *Nematostella*, the nuclear outer surface, comparable with the Nuclear Envelope (NE), expresses detectable levels of F-actin labeled with Lifeact-mTurquoise2. While the centrosomes form and position themselves adjacent to the nucleus a distinct and robust F-actin “flash” occurs and then the presumptive NE begins to deform and break down. When the spindle fibres connect between the two asters, the nuclear boundary is no longer visible by Lifeact-mTurquoise2 or phallacidin, suggesting it has been dissembled. As the two daughter cells form, F-actin is localized to the cell boundaries of each cell and F-actin returns to the nuclear boundary after the two cells separate.

Lifeact-mTurqoise2 as well as the untagged mVenus used as a control appears to be associated with the cytoplasmic position of centrosomes and mitotic spindles, however this phenomenon was not observed when using phallacidin. Although the levels of free cytosolic Lifeact-mtq2 and/or untagged mVenus are expected to be uniformly distributed in the cytoplasm, one potential explanation could be that the presence of yolk platelets reduces the effective volume for proteins. This effect will occur both for transparent and opaque yolk particles. When formation of tubulin fibres locally pushes yolk platelets away the effective volume that is accessible for fluorescent probes increases, and hence locally increases fluorescence that comes from free Lifeact-mTurqoise2 or free FP (untagged mVenus).

Compartmentalized F-actin along the nuclear boundary is not always clearly detectable by actin staining using common fixation methods. With the methods developed here, the rapid accumulation of F-actin prior to nuclear disassembly could be clearly and robustly observed. To date, this phenomena has been described in the sand dollar (Echinodermata) [38] and polychaete worms (Annelida) embryos, where it has been proposed that actin plays a role in accelerating nuclear envelope breakdown to facilitate faster cell cleavage [39]. The presence of NE breakdown in higher organism is still matter of debate, and is thought to have evolved as an extreme strategy to solve the problem of an increasing genome size, which is reflected by chromosome dimensions and number [40,41]. Semi-closed mitosis in which the NE remains almost intact has been observed in Bilateria such as *Caernorhabditis elegans* and *Drosophila melanogaste*r which have genome size of respectively 100 and 180 Mb [42], considerably smaller than *N. vectensis* (450 Mb) [43]. If open mitosis is a result of chromosomal dimension and genome size, then our observations using *N. vectensis* may support this hypothesis.

### Lifeact-mTurquoise2 a marker for cell morphology and specific cell types during development

Beyond the advantages of using Lifeact-mTurquoise2 to study *in vivo* cell cleavage in embryos, this technique also allows for visualization of F-actin throughout all stages of development. We observed F-actin in specific cellular structures such as microvilli, cnidocytes, ciliary cones in ciliated cells, and the plasma membrane. Furthermore, lifeact-mTurqoise2 allows for observation of real-time cell boundary dynamics and muscular contractions during animal movements in the adult polyps. Having the ability to trace cells *in vivo* will help validate modeling studies of cell mechanics [44].

## Conclusions

Methods developed in this manuscript provide an *in vivo* technique to follow cell cleavage during embryogenesis in *Nematostella vectensis*. The fluorescent probe, Lifeact-mTurquoise2, utilized in this manuscript provide a means to visualize specific cell types and structures that contain F-actin during development. One striking observation present during cellular cleavage was a ‘flash’ of Lifeact prior to nuclear disassembly. The presence of nuclear disassembly suggests that cell division in *Nematostella vectensis* goes through open mitosis, similar to many bilaterian taxa. The techniques developed in this study make *Nematostella* a more attractive model system for comparative developmental and cellular biology.

## Additional files

Additional file 1: Video S1. The process of cell division during embryonic development. Blastula stage embryos labeled with Lifeact-mTurquoise2 (cyan) and EB1-mVenus (yellow). Video subset is the same embryo visualized with phase contrast microscopy. Additional file 2: Video S2. The process of cell division during embryonic development. The same image series as Additional file 1: Video S1 but image series for Lifeact-mTurquoise2 (left, cyan) is separated from EB1-mVenus (right, yellow). Additional file 3: Video S3.
**A,B –** A “flash” of Lifeact-mTurquoise2 protein can be seen during cytokinesis, in particular breakdown of the nuclear envelop. **A)** Live image video of cells dividing during early embryonic development. Embryos are labeled with Lifeact-mTurquoise2 and show a flash of actin as the nuclear boundary breaks down prior to prometaphase. **B)** Cropped window from the same time series shows a full cycle of cell division. The intensity of the images at *t* =19.5-22.0 min, were slightly increased because the plane of focus changed. Additional file 4: Figure S1.
**A)** Variation among nuclear envelope folding in dividing cells. Eight different image stacks showing variation among the sequence of events immediately before and after nuclear envelop disassembly in eight different cells. The stages are about 30 seconds apart. **B)** Time series of control embryos during cellular cleavage using Lifeact-mTurquoise2 and mVenus. This control experiment shows specific localization of Lifeact at the cell boundary and is significantly lower for mVenus. **C)** Relative fluorescence increase of lifeact-mTurquoise2 at the cell cortex of dividing cells. Average line scan profiles perpendicular to the plasma membrane were measured at nuclear disassembly (red) and just prior to cleavage (blue and grey lines), individual profiles were subsequently normalized with the peak value of the profile from the earlier reference time point (grey, *n* =12). Only cell boundary regions were quantified that did not have an adjacent cell that was dividing. Although variable, the profiles just prior to cleavage on average (blue) show an increased fluorescence level with respect to the average profile measured at the time of nuclear disassembly (red). The light-colored lines represent the 95% confident intervals, which were obtained using bootstrapping based on 1000 bootstrap samples using sampling with replacement. **D)** Lifeact-mTurquoise2 ‘flash’ at the nuclear boundary. The grey profiles represent the normalized fluorescence perpendicular to the nuclear boundary going from nucleoplasm (left) to the cytoplasm (right). The profiles were measured just prior to nuclear disassembly and subsequently normalized with respect to the fluorescence in the nucleoplasm (*n* =16 cells and 3 different embryos). The green profile representing the average profile shows a 2-fold fluorescence increase. The light-colored lines represent the 95% confident intervals, which were obtained using bootstrapping based on 1000 bootstrap samples using sampling with replacement. Additional file 5: Video S4. Lifeact-mTurquoise2 localizes to cell and nuclear boundaries during division. A second video showing the breakdown of the nuclear boundary in relation to cell division. Additional file 6: Video S5. Microvilli at cellular boundary labeled with Lifeact-mTurquoise2. This video shows microvilli swirling erratically at the cellular junction of two cells. Additional file 7: Video S6. Lifeact-mTurquoise2 is a useful marker for visualization of cellular movement. This video shows cells transforming from a loose aggregate of cells into a more compact embryo. Additional file 8: Video S7. Concentrated Lifeact-mTurquoise2 protein is found along regions of contraction during peristalsis in juvenile polyps. Polyp labeled with Lifeact-mTurquoise2 from injection during embryonic development. This video shows the localization of Lifeact-mTurquoise2 around the site of contraction during peristaltic movement. Additional file 9: Figure S2. Long exposure time-lapse microscopy causes minimal bleaching and toxicity. **A)** Z-stack confocal image series of a Lifeact-mTurquoise2 injected embryo showing the depth of visible from the outer surface to approximately half way (75 microns) into the embryo. **B)** Surface image of a developing Lifeact-mTurquoise2 injected embryo over a four hour period of time. This embryo was exposed to six different z-plane scans per time-point (every 10 minutes) totaling 144 scans and exhibited little bleaching or toxicity.
